# Die neuen Reanimationsleitlinien 2021 und der hohe Stellenwert der Laienreanimation

**DOI:** 10.1007/s00103-022-03557-4

**Published:** 2022-06-20

**Authors:** Lina Horriar, Nadine Rott, Bernd W. Böttiger

**Affiliations:** Klinik für Anästhesiologie und Operative Intensivmedizin, Universitätsklinikum Köln, Kerpener Str. 62, 50937 Köln, Deutschland

**Keywords:** Laienreanimation, Wiederbelebungstrainings, Lebensrettende Systeme, BIG FIVE, Herz-Kreislauf-Stillstand, Lay resuscitation, CPR training, Systems Saving Lives, BIG FIVE, Cardiac arrest

## Abstract

Die Wiederbelebung durch Laien ist eine der wichtigsten Maßnahmen, um die Überlebensrate von Patientinnen und Patienten nach außerklinischem Herz-Kreislauf-Stillstand zu erhöhen. Während in anderen europäischen Ländern, vor allem in Skandinavien, Laienreanimationsquoten von über 80 % erreicht werden, liegt die Quote in Deutschland nur bei rund 40 %. Die vom European Resuscitation Council aktualisierten Reanimationsleitlinien 2021 messen den *lebensrettenden Systemen* eine zentrale Bedeutung bei und legen dabei einen Fokus auf die Wiederbelebung durch Laien. Die *lebensrettenden Systeme* betonen das Zusammenspiel zwischen allen an der Überlebenskette beteiligten Akteurinnen und Akteuren. So wird auch die Verbindung von Rettungsdienst und der Allgemeinbevölkerung konkretisiert.

Angelehnt an die *BIG-FIVE*-Überlebensstrategien nach Herz-Kreislauf-Stillstand werden 5 zentrale Strategien erläutert, mit denen die größte Verbesserung des Überlebens erreicht werden kann. Darunter fallen 1) die Erhöhung der Laienreanimationsquote durch Kampagnen und die schulische Ausbildung in Wiederbelebung *KIDS SAVE LIVES*, 2) die Implementierung der Telefonreanimation in Leitstellen, 3) Ersthelfersysteme, 4) die flächendeckende Advanced-Life-Support-Versorgung und 5) spezialisierte Kliniken, sogenannte Cardiac Arrest Centers, nach Herz-Kreislauf-Stillstand.

## Abstract (en)

Lay resuscitation is one of the most important measures to increase the survival rate of patients after out-of-hospital cardiac arrest. While European countries, and especially Scandinavian countries, achieve lay resuscitation rates of over 80%, the rate in Germany is only around 40%. The 2021 Resuscitation Guidelines updated by the European Resuscitation Council give special weight to *Systems Saving Lives* and focus on resuscitation by laypersons. The *Systems Saving Lives* emphasize the interplay between all actors involved in the chain of survival and thereby specify the link between the emergency service and the general population.

Based on the *BIG FIVE* survival strategies after cardiac arrest, five key strategies are outlined that can achieve the greatest improvement in survival. These are (1) increasing lay resuscitation rates through campaigns and *KIDS SAVE LIVES* school-based resuscitation training, (2) implementing telephone resuscitation in dispatch centers, (3) first responder systems, (4) advanced life support, and (5) specialized *cardiac arrest centers*.

## Einleitung

In Deutschland erleiden jährlich mehr als 70.000 Menschen einen Herz-Kreislauf-Stillstand [1]. In Europa ist dieser die dritthäufigste Todesursache [2]. Der wichtigste Schritt in der Überlebenskette Betroffener ist die Wiederbelebung durch Laien. Bis zum Eintreffen des Rettungsdienstes nach dem Kollaps und Absetzen eines Notrufs vergehen in Deutschland durchschnittlich 9 min [3]. Dabei zählt jede Minute, die das Gehirn ohne Sauerstoffzufuhr bleibt, denn bereits nach 3–5 min werden lebenswichtige Strukturen irreparabel zerstört. In Deutschland lag die Laienreanimationsquote im Jahr 2020 bei 40 %. Sie hat sich in den letzten Jahren zwar bereits deutlich gesteigert, ist aber dennoch niedrig im Vergleich mit anderen europäischen Ländern, in denen Quoten von 70–80 % erreicht werden [4]. Mit einer weiteren Erhöhung der Laienreanimationsquote auf ein solches Niveau wird eine Verdreifachung des Überlebens erwartet, sodass hierzulande jedes Jahr Tausende Menschenleben zusätzlich gerettet werden können [5, 6].

Der European Resuscitation Council (ERC) aktualisierte 2021 die Reanimationsleitlinien [7] basierend auf dem Konsens des International Liaison Committee on Resuscitation (ILCOR; [8]). Dieser Beitrag gibt einen Überblick über das Kapitel „Lebensrettende Systeme“ der aktualisierten ERC-Reanimationsleitlinien, welches die 2020 veröffentlichten BIG-FIVE-Überlebensstrategien nach Herz-Kreislauf-Stillstand [9] und damit vor allem die Laienreanimation und Ersthelfersysteme unterstreicht. Darüber hinaus werden Maßnahmen vorgestellt, die ergriffen werden müssen, um die Laienreanimationsquote und somit das Überleben auch hierzulande nachhaltig zu verbessern.

## Lebensrettende Systeme laut ERC-Reanimationsleitlinien

Die 2021 aktualisierten Reanimationsleitlinien des ERC geben dem Kapitel „Lebensrettende Systeme“ (Systems Saving Lives) eine zentrale Bedeutung und rücken wichtige Themen, die das Bewusstsein für Laienreanimation steigern, deutlich in den Vordergrund. Das Zielpublikum sind die Politik, das Management von Gesundheits- und Bildungssystemen, Angehörige der Gesundheitsberufe, Lehrerinnen und Lehrer, Schülerinnen und Schüler, Studierende und Laien [10]. Das Kapitel zeigt auf, dass ein Zusammenspiel vieler verschiedener Faktoren notwendig ist, um das Überleben von Patientinnen und Patienten mit Herz-Kreislauf-Stillstand weltweit bestmöglich zu verbessern [9]. Als Konzept der *lebensrettenden Systeme* wird der Zusammenhang zwischen den verschiedenen beteiligten Akteurinnen und Akteuren in der Überlebenskette als Ansatz auf Systemebene verdeutlicht. Das Ziel liegt in der Verbesserung der Überlebensrate eines Herz-Kreislauf-Stillstands außerhalb des Krankenhauses und/oder im Krankenhaus („out-of-hospital cardiac arrest“, OHCA und „in-hospital cardiac arrest“, IHCA). Erläutert werden 5 zentrale Strategien (BIG FIVE), mit denen die größte Verbesserung des Überlebens erreicht werden kann. Diese sind:

1. die Erhöhung der Laienreanimationsquote durch Aktionstage und Kampagnen wie den *World Restart a Heart Day* (WRAH) oder auch die Ausbildung von Schülerinnen und Schülern in Wiederbelebung *KIDS SAVE LIVES*,
2. die Implementierung der Telefonreanimation (T-CPR) in allen Leitstellen,
3. flächendeckende Ersthelfersysteme,
4. die flächendeckende Advanced-Life-Support(ALS)-Versorgung und
5. spezialisierte Kliniken (*Cardiac Arrest Centers,* CAC) zur Versorgung von Patientinnen und Patienten nach OHCA.

Im Folgenden werden die einzelnen Strategien der lebensrettenden Systeme näher erläutert. Zentral ist hierbei die Erhöhung des Bewusstseins für Laienreanimation durch Reanimationstrainings, der Aufbau von Communitys unter Einbindung neuer Technologien, die flächendeckende Implementierung der T‑CPR in Leitstellen sowie die Weiterversorgung von Patientinnen und Patienten in CAC.

## Punkt 1: Erhöhung der Laienreanimationsquote durch Aktionstage und Kampagnen sowie die Schülerausbildung in Wiederbelebung

**Figure 1:**
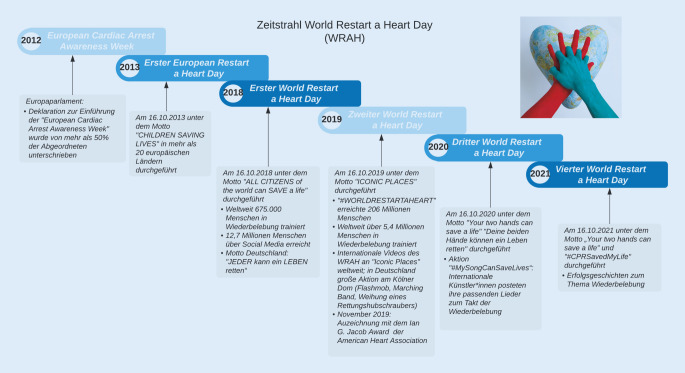

Europa- und weltweite Aktionstage wie der *European Restart a Heart Day* und der WRAH sollen mit jährlich wechselnden Mottos und unterschiedlichen Aktionen die Allgemeinbevölkerung für das Thema Herz-Kreislauf-Stillstand sensibilisieren und das Bewusstsein für die Wichtigkeit der Laienreanimation erhöhen [9]. Die Reanimationsleitlinien fordern, dass sich Regierungen und lokale Behörden am WRAH beteiligen und darüber viele Bürgerinnen und Bürger in Wiederbelebungsmaßnahmen schulen [10]. Der erste *European Restart a Heart Day* fand 2013 unter dem Motto *Children Saving Lives* statt. Beim ersten weltweiten WRAH am 16.10.2018 unter dem Motto *ALL CITIZENS of the world can SAVE a life – JEDER kann ein LEBEN retten* konnten bereits 675.000 Menschen in Wiederbelebungsmaßnahmen trainiert und 12,7 Mio. über Social Media erreicht werden [11]. Im folgenden Jahr konnten neben erfolgreichen Aktionen wie öffentlichen Reanimationstrainings mit insgesamt 5,4 Mio. Menschen [12] und Flashmobs auch mehr als 206 Mio. Menschen durch den Hashtag *#worldrestartaheart* via Social Media erreicht werden [12]. Bedingt durch die COVID-19-Pandemie wurden digitale Formate 2020 und 2021 ausgebaut. So posteten z. B. bei der Social-Media-Kampagne *#MySongCanSaveLives* zum WRAH 2020 nationale und internationale Künstlerinnen und Künstler ihre zum Takt der Wiederbelebung passenden Lieder in den sozialen Netzwerken und erlangten dadurch vor allem auch bei der jüngeren Bevölkerung große Aufmerksamkeit [13]. 2021 rief die Aktion *#CPRSavedMyLife* dazu auf, Geschichten von eigens erlebter Wiederbelebung sowie selbst durchgeführter Reanimationen zu teilen. Überlebende posteten Selfies mit einem Schild, auf dem ihr Name, Alter und *#CPRSavedMyLife* stand. Ziel war es zu verdeutlichen, dass jede Person in eine Situation gelangen kann, in der Hilfe benötigt wird. Die Kampagne erreichte mehr als 194 Länder und 200 Mio. Menschen (Abb. 1; [19]).

Deutschland hat im Vergleich zu anderen europäischen Ländern eine deutlich geringere Laienreanimationsquote. [4]. Dabei fällt besonders auf, dass Länder mit hohen Laienreanimationsquoten Wiederbelebungstrainings schon im Schulkindalter starten. Basierend auf Daten aus Dänemark, wo eine Steigerung der Laienreanimationsquote die Überlebensrate der Patientinnen und Patienten signifikant verbessert hat [6], ist es denkbar, mit ähnlich hohen Quoten in Deutschland jährlich bis zu 10.000 Menschenleben zusätzlich retten zu können [14]. Für eine dauerhafte Erhöhung der Laienreanimationsquote ist ein Beginn entsprechender Schulungen schon bei den Jüngsten notwendig. Hierzu positionierte sich u. a. auf Initiativen des Deutschen Rates für Wiederbelebung (German Resuscitation Council; GRC) schon 2014 die Kultusministerkonferenz zur Einführung des Wiederbelebungsunterrichts an Schulen [15]. Unter dem Projekt *KIDS SAVE LIVES* sollen dazu ab der 7. Klasse jährlich 2 h für die Ausbildung von Schülerinnen und Schülern aufgewendet werden, z. B. im Sport- oder Biologieunterricht sowie im Rahmen von Projekttagen. Das Training kann entweder durch speziell geschultes Lehrpersonal der eigenen Schule, Medizinstudierende, medizinisches Hilfspersonal oder Ärztinnen und Ärzte durchgeführt werden [15]. Gemäß den Prinzipien des ERC wird für *KIDS SAVE LIVES* eine Kombination von Theorie inklusive virtuellen Lernens und praktischer Anwendung empfohlen. Der Fokus liegt dabei auf den wichtigsten Schritten *PRÜFEN-RUFEN-DRÜCKEN*. Weiterhin sollen die Schulkinder ermutigt werden, als Hausaufgabe ihr Wissen an ihr Umfeld, z. B. Eltern, Geschwister und andere Verwandte, weiterzugeben und somit als Multiplikatoren zu dienen. Studien zeigen, dass ein Start des Trainings um das 12. Lebensjahr ideal, aber auch ein deutlich früherer Zeitpunkt möglich ist [16]. Dafür benötigt es ein landesweites Programm von Politik, Kultus- und Schulministerien für die Ausgestaltung und Implementierung des Wiederbelebungstrainings [17].

Weiterhin können Wiederbelebungstrainings auch in Hochschuleinrichtungen angeboten werden, insbesondere für Lehr- und Gesundheitsberufe. Passend dazu startete im September 2021 die Kampagne *#ichrettedeinleben* der Initiative *Wir beleben Deutschland wieder* und u. a. des GRC. Auf den Kampagnenplakaten zeigten sich professionelle Helferinnen und Helfer gemeinsam mit einem „Schülerzwilling“ unter dem Titel *Ich rette dein Leben – Rettest du meins?* (https://ichrettedeinleben.de/). Bereits in den ersten 3 Wochen wurden über 50.000 Unterschriften für eine Petition zu einem bundesweit verpflichtenden Wiederbelebungsunterricht für Schülerinnen und Schüler ab der 7. Klasse gesammelt. Durch die Unterstützung zahlreicher Influencerinnen und Influencer erzielten die Kampagne und das Thema eine große mediale Aufmerksamkeit. In der zweiten Runde der Kampagne konnten die Plakate aus den sozialen Medien ausgedruckt und u. a. in die Landeshauptstädte getragen werden, um somit Mitglieder der Parteispitzen sowie zahlreiche Gesundheits- und Bildungspolitikerinnen und -politiker auf Bundes- und Landesebene um Unterstützung zu bitten.

Durch die beschriebenen Aktionen und Kampagnen konnte die Laienreanimationsquote von ca. 16 % im Jahr 2008 [18] nach Angaben des Deutschen Reanimationsregisters auf rund 40 % im Jahr 2020 gesteigert werden [4].

## Punkt 2: Implementierung der T-CPR in Leitstellen

Die flächendeckende Implementierung der T‑CPR in Rettungsdienstleitstellen ist eine weitere Maßnahme zur Erhöhung der Laienreanimationsquote. Die neuen Leitlinien fordern daher bundesweit den Einsatz der T‑CPR durch die Disponentinnen und Disponenten. Bei einem Notruf erfolgt zunächst eine abfrageunterstütze Erkennung eines Herz-Kreislauf-Stillstands durch standardisierte Kriterien und Algorithmen. Anschließend wird telefonisch assistiert eine Laienreanimation für Ersthelfende durchgeführt. Schwerpunkt liegt dabei auf der Thoraxkompression [10]. Durch die Leitung der Disponentinnen und Disponenten können Hemmungen der Ersthelfenden reduziert und das Intervall, in dem keine Wiederbelebungsmaßnahmen unternommen werden, deutlich verkürzt werden. Studien weisen darauf hin, dass die Untätigkeit von Laien häufig vor allem durch Unsicherheiten bedingt ist [20]. Die Überlebensrate der Betroffenen kann durch eine angeleitete Wiederbelebung und somit Überbrückung der Zeitspanne bis zum Eintreffen des Rettungsdienstes verdoppelt werden [21]. 2021 führten dazu der GRC gemeinsam mit der Klinik für Anästhesiologie und Operative Intensivmedizin der Uniklinik Köln, der ADAC Stiftung und dem Fachverband Leitstellen e. V. eine Studie durch, an der 166 von 249 Rettungsleitstellen in Deutschland teilnahmen. Es zeigte sich, dass zwar alle teilnehmenden Leitstellen grundsätzlich auch T‑CPR durchführen, aber eine Umsetzungsquote von über 80 % bei Notrufen mit Herz-Kreislauf-Stillstand von nur weniger als der Hälfte der Leitstellen erreicht wird [22]. Laut dem Deutschen Reanimationsregister lag 2020 die telefonische Anleitung zur Reanimation in den Referenzstandorten bei nur 23,7 % [4]. Gründe für die niedrige Umsetzungsquote der T‑CPR in Leitstellen waren laut der Studie vor allem ein hohes parallel abzuarbeitendes Notrufaufkommen, personelle Engpässe sowie unzureichende Qualifikationen der Disponentinnen und Disponenten. Für eine erfolgreiche Implementierung sollten Schulungen der Mitarbeitenden und gesetzliche Vorgaben umgesetzt werden [22].

## Punkt 3: Ersthelfersysteme

Ersthelfersysteme (*First-Responder-Systeme)* bezeichnen Technologien, die die Bevölkerung in die Überlebenskette miteinbeziehen. Hierbei kommen z. B. Smartphone-Apps zum Einsatz, die mittels Ortungssystem oder Textnachricht Ersthelfende informieren, die sich in der Nähe einer Person mit OHCA befinden. Wie bei der T‑CPR ist das Ziel, die Zeit bis zur ersten Herzdruckmassage und ggf. Defibrillation zu verkürzen und damit das Überleben inklusive eines guten neurologischen Outcomes signifikant zu verbessern [10]. Gefördert werden soll gleichzeitig auch der Aufbau von ersthelfenden Communitys. Beteiligen können sich je nach System sowohl geschulte als auch ungeschulte Laien, Feuerwehrleute, Polizistinnen und Polizisten und Angehörige von Gesundheitsberufen. Der Ausbau von etablierten Systemen ist in Deutschland noch nicht flächendeckend umgesetzt. Es gibt zahlreiche ortsgebundene Systeme, die untereinander allerdings nicht kompatibel sind und einen unterschiedlichen Funktionsumfang aufweisen [23]. Eine flächendeckende Implementierung und eine stetige Weiterentwicklung sind auch hier im Sinne des Überlebens der Betroffenen dringend notwendig [10].

Im Kreis Gütersloh konnte beispielsweise im Rahmen des Modellprojektes *Mobile Retter* ein dichtes Ersthelfernetz ausgebaut werden. Die registrierten Personen wurden bei einem Notruf parallel zum Rettungsdienst über eine App alarmiert und zum Einsatzort navigiert. Bei 78 % der Einsatzübernahmen trafen die mobilen Retterinnen und Retter dort dann vor oder gleichzeitig mit dem Rettungsdienst ein [23].

Im Jahr 2018 führte der Verein Region der Lebensretter e. V. in Freiburg im Breisgau ein weiteres System ein, welches im Notfall mittels Smartphone-App registrierte, medizinisch geschulte Ersthelferinnen und Ersthelfer, die sich in der Nähe eines Unfallortes befinden, alarmiert. Lagen die Einsatzübernahmen im zweiten Halbjahr 2018 noch bei 30 % und die Anzahl an Helferinnen und Helfern bei 276, stiegen sie im ersten Halbjahr 2020 auf 49 % und 794. 2020 betrug die mittlere Eintreffzeit der Ersthelfenden 6,09 min, wodurch das reanimationsfreie Intervall erheblich verkürzt werden konnte [24].

Erwartet wird durch den Einsatz von Ersthelfersystemen eine 1,2- bis 2‑fache Verbesserung des Überlebens [9, 25]. Laut Jahresbericht des Reanimationsregisters für 2020 erfolgte bei 4,4 % aller Patientinnen und Patienten die Reanimation vor Eintreffen des Rettungsdienstes durch einen First Responder [4].

## Punkt 4: Flächendeckende ALS-Versorgung

Die ALS-Versorgung bezeichnet erweiterte lebensrettende Maßnahmen, die von professionellen Helferinnen und Helfern im Rahmen der Wiederbelebung von Notfallopfern durchgeführt werden [26]. Die begonnenen Basismaßnahmen zur Wiederbelebung (BLS) und die darauffolgende ALS-Versorgung greifen idealerweise ineinander [27]. Dabei werden präklinische von innerklinischen Ereignissen unterschieden. Zur ALS-Versorgung in präklinischen Situationen gehören die Alarmierung des Rettungsdienstes und Maßnahmen der Notärztinnen und Notärzte sowie des Rettungsdienstpersonals [28]. Studien zeigen eine Verdopplung des Überlebens in notarztgestützten Systemen [29]. Bei innerklinischen medizinischen Notfällen kommen für die ALS-Versorgung Response-Teams zum Tragen. Darunter fallen Medical-Emergency-Teams und das in der Notaufnahme sowie in der Versorgung von Patientinnen und Patienten qualifizierte Krankenhauspersonal. Zusammen mit Frühwarnscores, die das Risiko einer Verschlechterung zeigen, können die Häufigkeit eines IHCA sowie die Mortalität verringert werden [10].

## Punkt 5: Cardiac Arrest Center

**Figure 2:**
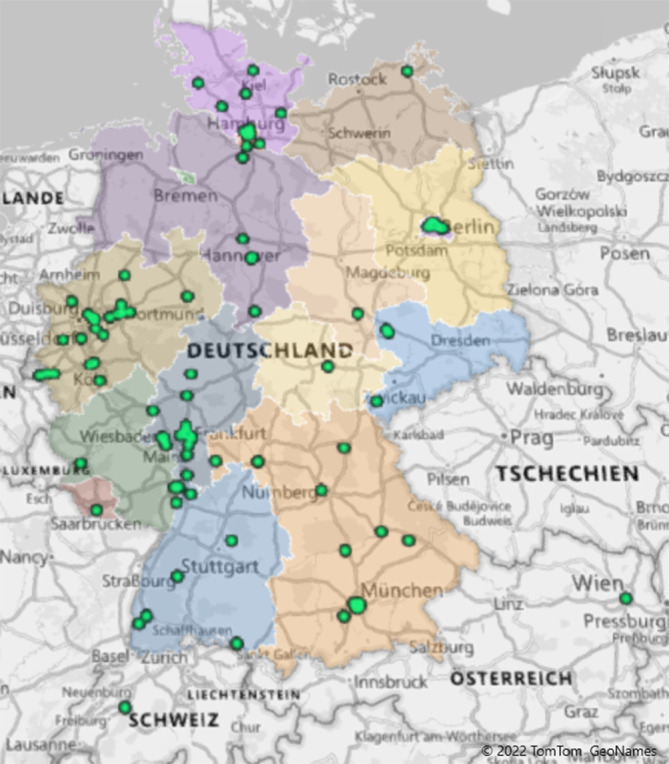

Nach einem OHCA sollten Patientinnen und Patienten bestenfalls in spezialisierten Kliniken, sogenannten Cardiac Arrest Centers (CAC), versorgt werden, in denen eine Verdopplung der Überlebensrate erwartet wird [9, 30, 31]. Die neuen Reanimationsleitlinien 2021 betonen daher noch einmal deutlich die Relevanz der CAC. Fehltransporte in nicht geeignete Krankenhäuser sollen möglichst vermieden werden. Die Prognose nach erfolgreicher Reanimation ist wesentlich abhängig von der Qualität, Spezialisierung, Fachkompetenz und Ausstattung der weiterbehandelnden Klinik. Um eine einheitliche Versorgungsqualität zu erreichen, wurden 2017 Kriterien für die CAC eingeführt. Unter dem Schirm des GRC entwickelte eine Arbeitsgruppe aus den Fachbereichen Anästhesiologie, Kardiologie und Intensivmedizin Basisanforderungen, die von der Deutschen Gesellschaft für Anästhesiologie und Intensivmedizin (DGAI), der Deutschen Gesellschaft für Kardiologie, Herz- und Kreislaufforschung (DGK) und der Deutschen Gesellschaft für Internistische Intensiv- und Notfallmedizin (DGIIN) konsentiert und in entsprechenden Fachzeitschriften publiziert wurden. Dabei zählen zu den wichtigsten Kriterien eine besondere Struktur für die Versorgung der Patientinnen und Patienten, die Sicherstellung einer adäquaten Prozessqualität mit Nachweis der Verwendung von Standard Operating Procedures sowie die Qualitätssicherung mit Nachweis einer standardisierten Erfassung des Behandlungsverlaufs bis zur Entlassung [32]. In Deutschland finden Auditierungen von CAC seit Ende 2018 statt, die ersten Kliniken konnten 2019 erfolgreich zertifiziert werden. Stand 2021 sind knapp 100 Krankenhäuser in 14 der 16 Bundesländer zertifiziert, Ziel ist eine flächendeckende Versorgung (Abb. 2). Auch im deutschsprachigen Ausland konnten bereits die ersten Kliniken zertifiziert werden [33]. Aufgrund weitreichender Erfahrungen aus dem Zertifizierungsprozess veröffentlichten der GRC und die DGK Mitte 2021 einen aktualisierten Kriterienkatalog [32]. Aktuell gibt es weiteren Forschungsbedarf zu den Effekten der Implementierung der CAC. Aufgrund der derzeitig nicht ausreichenden Evidenzlage sprechen die ERC-Leitlinien lediglich eine schwache Empfehlung zur Versorgung von Patientinnen und Patienten in CAC aus [10].

## Das Konzept der *lebensrettenden Systeme*

Im Konzept der *lebensrettenden Systeme* wird das Zusammenspiel vieler verschiedener Faktoren genannt, die in der Überlebenskette beteiligt sind. Dabei ist eine Vielzahl von Akteurinnen und Akteuren notwendig, um die Überlebensquoten von Betroffenen zu steigern. Die Maßnahmen der *BIG-FIVE*-Überlebensstrategien sind mit einer deutlichen Erhöhung des Überlebens assoziiert. So wird z. B. durch Projekte wie *KIDS SAVE LIVES* eine Verbindung zwischen Bevölkerung und Rettungsdienst hergestellt. Neben den oben beschriebenen Schulungsinhalten lernen die Schulkinder hier auch, den Rettungsdienst zu alarmieren. Letzterer kann dann ggf. im Rahmen der T‑CPR Anweisungen zu weiterem Vorgehen geben. Gleichzeitig sensibilisieren Kampagnen und Aktionstage die Allgemeinbevölkerung für das Thema Reanimation und Schülerinnen und Schüler geben ihr Wissen aus dem Unterricht innerhalb ihrer Familien weiter. Mithilfe von Ersthelfersystemen entstehen lokale Communitys mit dem Ziel, die Quote der Ersthelferinnen und Ersthelfer zu steigern und mehr Leben zu retten [10]. Die Steigerung der Laienreanimationsquote erhöht das Überleben um den Faktor 3 [5, 6]. Von T‑CPR, ALS-Teams und CAC wird eine Verdopplung des Überlebens erwartet [21, 29–34]. Der Ausbau von Ersthelfersystemen lässt den Faktor um 1,2 bis 2 steigen [9, 25, 35, 36].

## Fazit

Die *lebensrettenden Systeme* und insbesondere die Laienreanimation sind zentral wichtige Glieder in der Überlebenskette nach Herz-Kreislauf-Stillstand. Durch gezielte Förderung der einzelnen Strategien kann eine dauerhafte und deutliche Erhöhung der Überlebensrate von Patientinnen und Patienten mit OHCA erreicht werden. Hier sind insbesondere die Politik und auch einzelne Kommunen gefordert, flächendeckende Ersthelfersysteme kontinuierlich auszubauen sowie die Einführung eines verpflichtenden Reanimationsunterrichts für Schülerinnen und Schüler durchzusetzen. Erfreulicherweise konnte die Laienreanimationsquote, die in Deutschland 2008 nur bei 16 % lag, durch diverse Aufklärungsarbeiten bis 2021 auf ca. 40 % gesteigert werden [18]. Um diese Quote noch weiter zu steigern, sind zusätzliche Maßnahmen zur Förderung der Laienreanimation, klare und bundesweite gesetzliche Vorgaben sowie eine deutschlandweite Implementierung der *lebensrettenden Systeme* notwendig. Durch einen Ausbau von sich ergänzenden Maßnahmen, wie die Umsetzung eines verpflichtenden Wiederbelebungsunterrichts für Schulkinder, den Ausbau von etablierten Ersthelfersystemen oder die Durchführung der T‑CPR in Leitstellen, ist es denkbar, dass bis zu 10.000 Menschenleben jährlich zusätzlich gerettet werden können [14].
